# Supplementary material for: ntEdit: scalable genome sequence polishing
Source: Bioinformatics. 2019 May 16;35(21):4430–2. doi: 10.1093/bioinformatics/btz400 (PMC6821332; doi:10.1093/bioinformatics/btz400)
Supplement: btz400_Supplementary_Data [file btz400_supplementary_data.pdf]

Supplementary Data

Supplementary Methods

Description of ntHits

ntHits is a streaming algorithm and software tool for *de novo* repeat identification based on the statistical analysis of the kmer content profile of large-scale DNA sequencing data. It first generates the kmer coverage histograms of input datasets using the ntCard algorithm, an efficient streaming algorithm for estimating kmer coverage histograms (Mohamadi et al., 2017). From the obtained kmer coverage histogram, the repetitive kmers would present a long tail to the distribution of kmer coverage profile. ntHits identifies a threshold from the long tail of kmer profile distribution and outputs the highly repetitive kmers with frequency greater than the identified threshold. We have adapted ntHits (v0.0.1) to work with ntEdit by reporting a Bloom filter (--outbloom option) of non-error kmers. On controlled data, we ran ntHits with a set threshold (-c 1 on <20X coverages and –c 2 on ≥ 20X, indicating kmers with a coverage above 1 and 2, respectively). On the experimental human runs, ntHits ran on a 54X coverage Illumina read data in auto mode (nthits options --outbloom --solid -b 36 -k 35 -t 48), using a threshold set by the ntCard algorithm based on the corresponding kmer coverage histogram it computes (Fig. S4).

Data and run parameters on controlled experiments

To best assess ntEdit's correction performance we used pIRS v2.0.0 (Hu et al., 2012) to simulate *E. coli*, *C. elegans* and *H. sapiens* triplicate genome copies from their respective *E. coli* K12 MG1655 (U00096.3), *C. elegans* Bristol N2 (NC_003281.10) and *H. sapiens* (GRCh38) reference genomes, with substitution and indel rates of 0.001 and 0.0001, respectively (pirs diploid <genome.fa> -s 0.001 -d 0.0001). These were supplied to ntEdit (-f option) in lieu of draft assemblies (Fig. S2). Using the corresponding reference genomes, we simulated three sets of paired-end 100 bp reads with 0.1% error using Illumina error models at 10-50, 15-50 and 17-fold sequence coverages, respectively (pirs simulate -l 100 -x <coverage> -m 300 -e 0.001 --no-gc-bias --no-indels -B humNew.PE100.matrix.gz). We ran Pilon (v1.22 --changes --diploid --fix snps,indels) and GATK (v4.1.0.0) with bwa mem (v.0.7.17-r1188). GATK also ran with the help of samtools and bcftools (v1.9). The VCF file generated using bcftools was filtered to only include variants with IMF >= 0.7 and (reads supporting variant)/(total reads) >= 0.7 prior to polishing the input assembly with "gatk FastaAlternateReferenceMaker". Racon (v1.3.1), with Minimap2 (v2.15) and ntEdit (v1.1.0 -k 20-70 step 5 or 10, -i 4 -d 5 -x 5 -y 9) with ntHits (v0.0.1 -c 1 --outbloom) also ran on these datasets and the resulting assemblies were assessed with QUAST-LG v5.0.2, comparing the edited copies to their respective reference genome (Fig. S3). To speed up the Racon runs on the full *H. sapiens* genome, following the Minimap2 alignments of the simulated reads to the genome, the assembly, read alignments and reads were partitioned into 24 separate jobs; one per chromosome. Then, Racon polishing of each chromosome was run in parallel on a computing cluster (Intel Zeon E5-2650, 2.20 GHz, 384GB RAM, 48 threads). We also ran ntEdit iteratively on *E. coli*, *C. elegans* and *H. sapiens* at k35, k30, k25 and k40, k35, k30, k25 and k50, k45, k40, respectively.

Data and run parameters on experimental data

We polished NA12878 human genome drafts, including an assembly of 1) Pacific Bio sciences (PacBio) (Pendleton et al., 2015), 2) Nanopore (Jain et al., 2018) and 3) 10x Chromium linked (https://support.10xgenomics.com/de-novo-assembly/datasets/2.1.0/wfu) reads with ntEdit (several parameters – see Table S8), Racon (-u -w 500) and GATK (variant filtering same as

for controlled experiments) using Illumina reads for correction (downloaded from https://basespace.illumina.com public dataset : HiSeq 2500: TruSeq PCR-Free DNA 2x251 (NA12878), flowcell H00DDBCXX). In order to run Racon in a reasonable amount of time, we first aligned the full read set to each draft assembly with Minimap2, then split the draft assemblies, read alignments and reads into 20 partitions. Racon was run on each partition in parallel on a compute farm (Intel Zeon E5-2650, 2.20 GHz, 384GB RAM), with each job using 48 threads. We assessed the resulting, corrected assemblies, with BUSCO (Simão et al., 2015; v.2.0.1 lineage: euarchontoglires_odb9, eukaryota) to show both completeness and resolution of frameshift errors by reporting recovered gene products. Finally, to show scale, we ran ntEdit (-k50 -i1 -d1 -x5 -y9 -t 48) on the 20-Gbp interior (PG29-v4: Genbank GCA_000411955.5) and white (WS77111-v1: Genbank GCA_000966675.1) spruce genome pseudo haploid assemblies using DNA sequences from their corresponding, haploid tissue source (seed megagametophyte, SRA SRX5086197 and SRX5086196). GATK, ntHits, ntEdit, and Pilon, ran on a single CPU on *E. coli* and *C. elegans*. On controlled and experimental *H. sapiens* data, and spruce, all tools used 48 threads. Run time and memory usage was benchmarked on a CentOS 7 system with 128 CPU Intel(R) Xeon(R) CPU E7-8867 v3 @ 2.50GHz. Additional details are available at : http://www.bcgsc.ca/downloads/btl/ntedit/paper/

References:

Hu, X., et al. (2012) pIRS: Profile-based Illumina pair-end reads simulator. *Bioinformatics*. **28**. 1533-1535.

Jain, M., et al. (2018) Nanopore sequencing and assembly of a human genome with ultra-long reads. *Nat. Biotechnol*. **36**. 338–345.

Mohamadi, H., et al. (2017) ntCard: a streaming algorithm for cardinality estimation in genomics data. *Bioinformatics.* **33**. 1324-1330.

Pendleton, M., et al. (2015) Assembly and diploid architecture of an individual human genome via single-molecule technologies. *Nat. Methods.* **12.** 780–786.

Simão, R.M., et al. (2015) BUSCO: assessing genome assembly and annotation completeness with single-copy orthologs, *Bioinformatics.* 31. 3210–3212.

Supplementary Figures

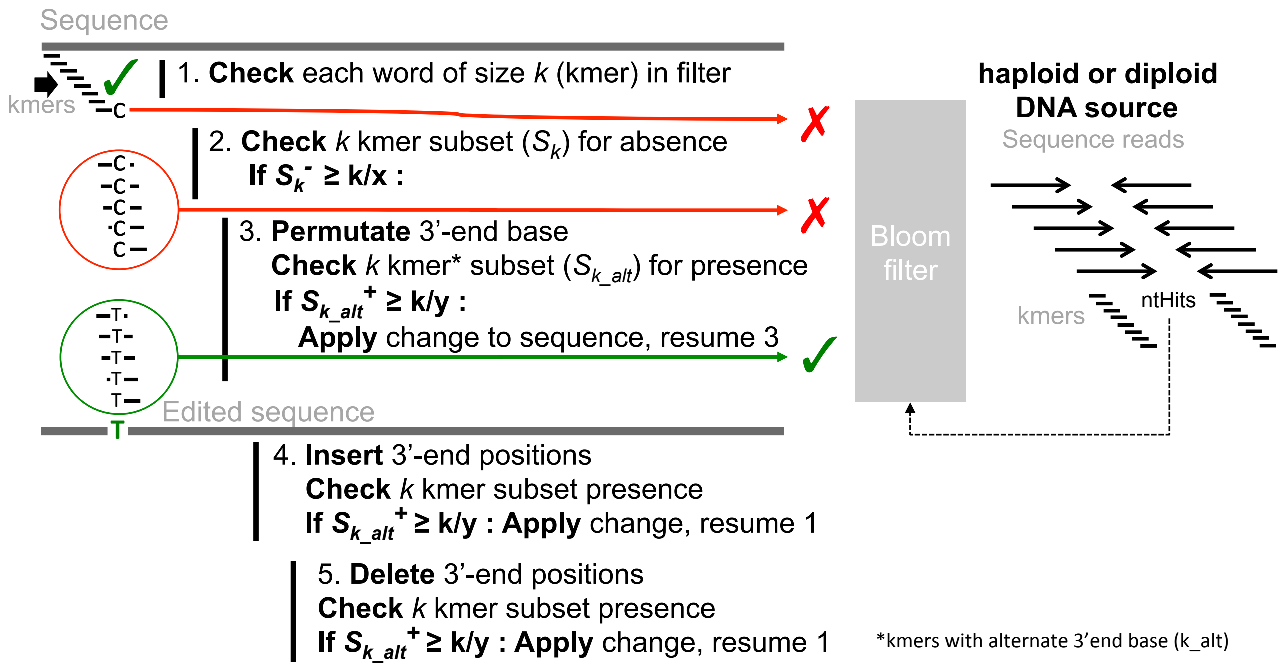

Supplemental Figure S1. ntEdit approach. Sequence reads are first shredded into kmers using ntHits, keeping track of kmer multiplicity. The kmers that pass coverage thresholds (ntHits, -c option builds a filter with kmers having a coverage higher than c) are used to construct a Bloom filter (BF). The draft assembly is supplied to ntEdit (-f option, fasta file), along with the BF (-r option) and sequences are read sequentially. Sequence strings are shredded into words of length k (kmers) at a specified value (-k option) matching that used to build the BF, and each kmer from 5' to 3' queries the BF data structure for presence/absence (step 1). When a kmer is not found in the filter, a subset (S_k) of overlapping k kmers (defined by k over three, k/3) containing the 3'-end base is queried for absence (step 2). The subset S_k, representing a subsampling of k kmers obtained by sliding over 3 bases at a time over k bases, is chosen to minimize the number of checks against the Bloom filter. Of this subset, when the number of **absent (-)** kmers matches or exceeds a threshold defined by S_k^- >= k/x (-x option), representing the majority of kmers in S_k, editing takes place (step 3 and beyond), otherwise step 1 resumes. In the former case, the 3'-end base is permuted to one of the three alternate bases (step 3), and the subset (S_{k_alt}) containing the change is assessed for Bloom filter **presence (+)**. When that number matches or exceeds the threshold defined by $S_{k\_alt}^+$ >= k/y (-y option), which means the base substitution qualifies, it is tracked along with the number of supported kmers and the remaining alternate 3'-end base substitutions are also assessed (ie. resuming step 3 until all bases inspected). If the edit does not qualify, then a cycle of base insertion(s) and deletion(s) of up to –i and –d bases begins (step 4, -i option and step 5, -d option, respectively). As is the case for the substitutions, a subset of k kmers containing the indel change is tested for presence. If there are no qualifying changes, then the next alternate 3'-end base is inspected as per above; otherwise the change is applied to the sequence string and the next assembly kmer is inspected (step 1). The process is repeated until a qualifying change or until no suitable edits are found. In the latter case, we go back to step 1. When a change is made, the position on the new sequence is tracked, along with an alternate base with lesser or equal k kmer subset support, when applicable. Currently, ntEdit only tracks cases when edits are made (steps 3-5), and does not flag unedited, missing draft kmers (steps 1-2).

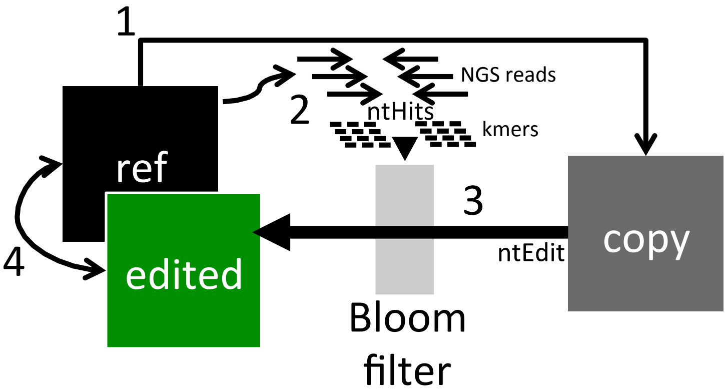

Supplemental Figure S2. Testing procedure on controlled data. To test against a ground truth, we made three copies of the *E. coli* K12 MG1655 (U00096.3), *C. elegans* Bristol N2 (NC_003281.10) and *H. sapiens* (GRCh38) reference genomes with pIRS (v2.0.0 pirs diploid <genome.fa> -s 0.001 -d 0.0001), tracking the coordinates and edits made (step 1). Using the reference genomes, we simulated PE100 reads using an Illumina error model at 0.1% error rate, and several fold coverages (10, 15, 20, 30, 40, 50) (pirs simulate -l 100 -x <COVERAGE> -m 300 -e 0.001 --no-gc-bias --no-indels -B humNew.PE100.matrix.gz) and built Bloom filters for each with ntHits (-c 1) (step2). Using the copy, we ran ntEdit/comparators in conjunction with the reference-derived reads (step 3). The resulting draft assembly was compared to the original reference genome and copy with QUAST (v5.02), tracking the number of mismatches and indels per 100kbp (step4) (tables S1-S3). Additionally, we made statistical measures of the performance. We report those values in tables S4, S5, and S6 (below).

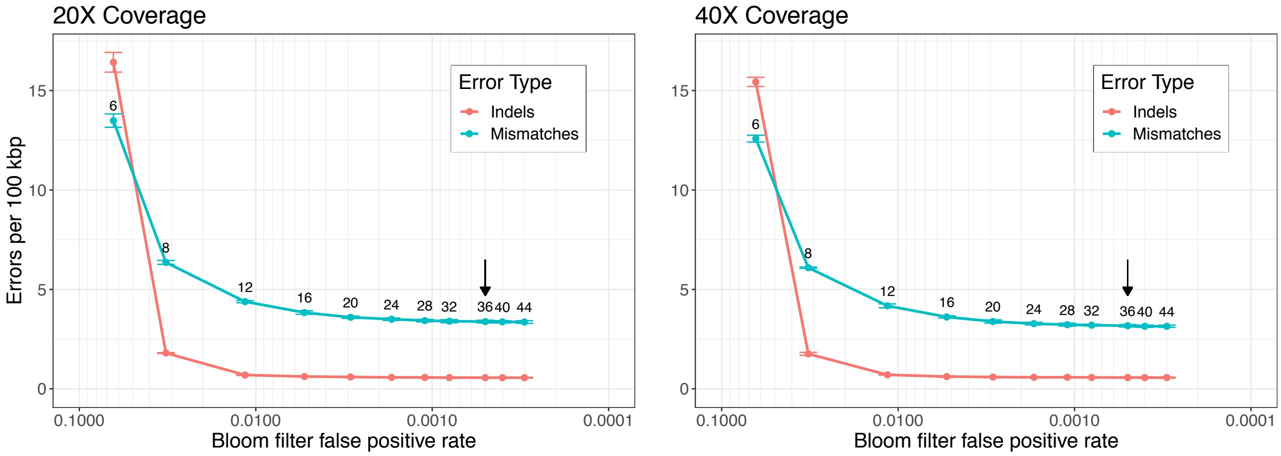

Supplemental Figure S3. Effect of Bloom filter false positive rate on ntEdits predictions. On the *C. elegans* controlled experiments (20X and 40X coverage data, left and right panels) and in triplicate experiments, we used ntHits to output Bloom filters (-c 1 --outbloom –k 35), controlling their bit sizes (-b option, values indicated on the plot) but using the same set of reads. The Bloom filter false positive rate (FPR) was calculated as FPR=(pop. count/bit size)^{hashes}. We ran ntEdit (-k 35 –i 4 –d 5 –x 5 –y 9) using each Bloom filter as input and the resulting, edited assembly was assessed by QUAST. By default, ntHits outputs Bloom filters with a FPR <0.001 (the ones used in our study were consistently set at b=36, FPR≈0.0005, black arrows). In that range, the Bloom filter FPR has little effect on the performance of ntEdit.

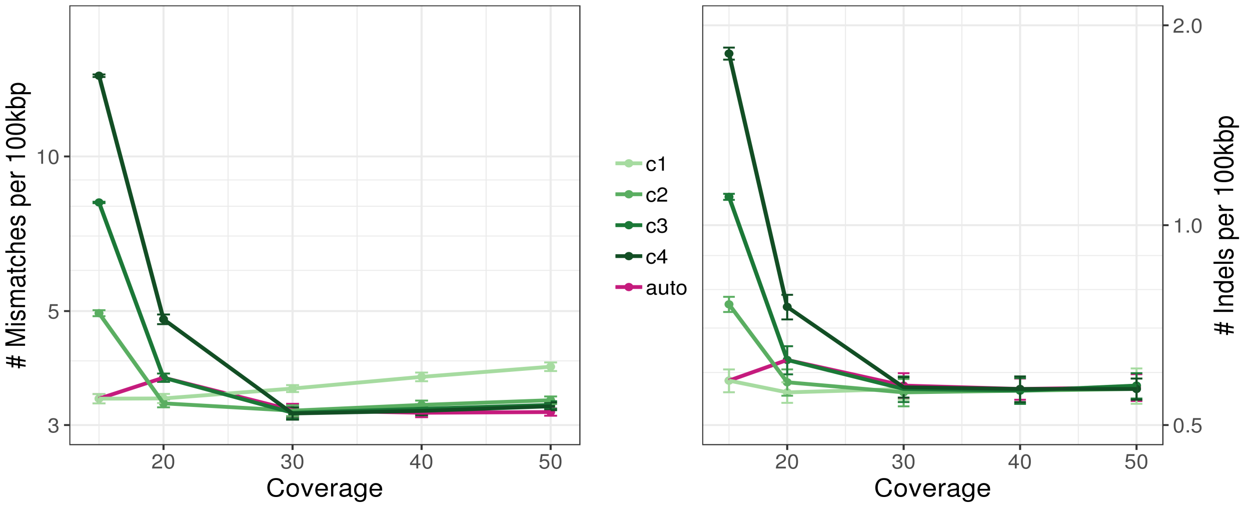

Supplemental Figure S4. Effect of the ntHits threshold c parameter. In separate experiment, we ran ntHits using hard thresholds of -c (1,2,3,4) to discard kmers (at those coverage and lower) and build a Bloom filter with the remaining kmers (nthits options -c 1 -b 36 --outbloom -k 35 -t 48). We also ran ntHits in auto mode, where the threshold for discarding erroneous kmers is set automatically by ntCard based on the kmer coverage histogram (nthits options --outbloom --solid -b 36 -k 35 -t 48). We then ran ntEdit using each Bloom filter as input and the resulting edited assembly was assessed with QUAST (ntEdit options -k 35 -d 5 -i 4 –x 5 –y 9). We here show the effect (mismatches and indels per 100kbp, left and right panels, respectively) of setting these thresholds on the *C. elegans* controlled data.

Supplemental Tables

Supplemental table 1. QUAST and resource benchmark results for controlled *E. coli* dataset.

Assembly	Cover-age	Mismatches per 100kbp	Indels per 100kbp	Time (min.)	Peak memory (Mb)
Baseline	N/A	99.81 +/- 0.85	9.99 +/- 0.07	N/A	N/A
GATK	10	1.49 +/- 0.11	4.53 +/- 0.13	1.07 +/- 0.05	599.77 +/- 9.48
	15	1.45 +/- 0.19	4.43 +/- 0.12	1.52 +/- 0.12	595.40 +/- 4.51
	20	1.38 +/- 0.16	4.40 +/- 0.06	1.92 +/- 0.22	601.49 +/- 15.82
	30	1.36 +/- 0.17	4.37 +/- 0.05	2.70 +/- 0.27	593.11 +/- 0.98
	40	1.36 +/- 0.14	4.32 +/- 0.13	3.53 +/- 0.45	592.59 +/- 4.36
	50	1.41 +/- 0.16	4.29 +/- 0.10	4.50 +/- 0.84	663.18 +/- 0.72
Racon	10	3.51 +/- 0.28	0.58 +/- 0.19	1.08 +/- 0.09	494.14 +/- 8.86
	15	1.57 +/- 0.17	0.35 +/- 0.05	1.62 +/- 0.23	720.66 +/- 11.55
	20	1.15 +/- 0.07	0.29 +/- 0.07	2.11 +/- 0.25	934.52 +/- 7.26
	30	1.08 +/- 0.19	0.24 +/- 0.09	3.05 +/- 0.14	1445.02 +/- 7.68
	40	0.98 +/- 0.17	0.27 +/- 0.10	4.30 +/- 0.55	1886.18 +/- 30.53
	50	1.07 +/- 0.11	0.20 +/- 0.06	5.32 +/- 0.71	2378.52 +/- 22.27
Pilon	10	11.73 +/- 0.12	1.24 +/- 0.16	10.27 +/- 0.10	2577.22 +/- 244.04
	15	2.33 +/- 0.25	0.24 +/- 0.06	9.21 +/- 0.70	2518.02 +/- 110.24
	20	1.51 +/- 0.18	0.17 +/- 0.02	6.53 +/- 0.82	2524.06 +/- 124.19
	30	1.26 +/- 0.21	0.16 +/- 0.03	4.68 +/- 1.06	2594.91 +/- 199.33
	40	1.19 +/- 0.18	0.16 +/- 0.01	4.22 +/- 0.67	2550.86 +/- 219.62
	50	1.08 +/- 0.17	0.15 +/- 0.02	4.85 +/- 0.24	2392.18 +/- 217.45
ntEdit k20	10	2.50 +/- 0.57	0.40 +/- 0.07	0.11 +/- 0.00	515.48 +/- 0.06
	15	1.64 +/- 0.21	0.37 +/- 0.05	0.13 +/- 0.00	515.46 +/- 0.03
	20	1.58 +/- 0.23	0.34 +/- 0.03	0.14 +/- 0.01	515.46 +/- 0.02
	30	1.56 +/- 0.19	0.34 +/- 0.04	0.19 +/- 0.01	515.50 +/- 0.02
	40	1.59 +/- 0.27	0.34 +/- 0.04	0.22 +/- 0.01	515.53 +/- 0.03
	50	1.59 +/- 0.22	0.34 +/- 0.04	0.26 +/- 0.02	515.49 +/- 0.00
ntEdit k25	10	2.77 +/- 0.83	0.44 +/- 0.09	0.11 +/- 0.01	515.47 +/- 0.03
	15	1.56 +/- 0.19	0.34 +/- 0.05	0.13 +/- 0.00	516.77 +/- 2.30
	20	1.50 +/- 0.18	0.32 +/- 0.05	0.14 +/- 0.00	515.48 +/- 0.00
	30	1.50 +/- 0.25	0.31 +/- 0.04	0.17 +/- 0.01	515.52 +/- 0.04
	40	1.46 +/- 0.18	0.33 +/- 0.05	0.21 +/- 0.00	515.43 +/- 0.06
	50	1.53 +/- 0.22	0.32 +/- 0.05	0.24 +/- 0.02	515.50 +/- 0.01
ntEdit k30	10	4.36 +/- 1.48	0.58 +/- 0.12	0.11 +/- 0.01	515.48 +/- 0.00
	15	2.31 +/- 0.29	0.42 +/- 0.04	0.13 +/- 0.01	516.80 +/- 2.31
	20	2.25 +/- 0.27	0.41 +/- 0.04	0.14 +/- 0.01	515.47 +/- 0.01
	30	2.20 +/- 0.26	0.40 +/- 0.05	0.17 +/- 0.01	516.85 +/- 2.32
	40	2.19 +/- 0.24	0.42 +/- 0.03	0.21 +/- 0.01	515.43 +/- 0.10
	50	2.23 +/- 0.27	0.42 +/- 0.05	0.23 +/- 0.02	515.47 +/- 0.04
ntEdit k35,k30, k25	10	2.26 +/- 0.23	0.39 +/- 0.05	0.34 +/- 0.01	516.84 +/- 2.30
	15	1.45 +/- 0.20	0.32 +/- 0.03	0.39 +/- 0.02	515.48 +/- 0.03
	20	1.43 +/- 0.21	0.31 +/- 0.05	0.44 +/- 0.06	515.50 +/- 0.03
	30	1.40 +/- 0.21	0.31 +/- 0.05	0.51 +/- 0.00	515.51 +/- 0.03
	40	1.40 +/- 0.21	0.31 +/- 0.05	0.60 +/- 0.01	515.51 +/- 0.05
	50	1.42 +/- 0.22	0.31 +/- 0.05	0.69 +/- 0.06	515.45 +/- 0.07

Supplemental table 2. QUAST and resource benchmark results for controlled *C. elegans* dataset.

Assembly	Cover-age	Mismatches per 100 kbp	Indels per 100 kbp	Time (min.)	Peak memory (Mb)
Baseline	N/A	99.82 +/- 0.15	9.91 +/- 0.05	N/A	N/A
GATK	15	1.91 +/- 0.04	4.81 +/- 0.01	48.40 +/- 2.47	4878.71 +/- 3481.10
	20	1.85 +/- 0.04	4.79 +/- 0.01	72.84 +/- 9.83	8473.77 +/- 2345.65
	30	1.80 +/- 0.05	4.75 +/- 0.03	93.13 +/- 11.65	6949.83 +/- 816.23
	40	1.77 +/- 0.05	4.73 +/- 0.01	114.98 +/- 2.50	8236.92 +/- 2000.48
	50	1.78 +/- 0.04	4.71 +/- 0.03	145.52 +/- 11.03	7894.65 +/- 1434.75
Pilon	15	2.90 +/- 0.07	0.36 +/- 0.01	322.63 +/- 8.63	24986.26 +/- 1367.40
	20	1.89 +/- 0.05	0.25 +/- 0.02	158.40 +/- 6.10	28191.86 +/- 2538.10
	30	1.52 +/- 0.08	0.21 +/- 0.02	134.97 +/- 2.62	24005.51 +/- 1584.47
	40	1.38 +/- 0.09	0.19 +/- 0.01	126.87 +/- 8.55	25133.68 +/- 468.06
	50	1.30 +/- 0.09	0.18 +/- 0.01	120.07 +/- 0.85	26211.87 +/- 853.61
Racon	15	2.03 +/- 0.10	0.57 +/- 0.03	46.87 +/- 4.40	15288.99 +/- 133.17
	20	1.63 +/- 0.07	0.52 +/- 0.04	65.53 +/- 6.97	20127.11 +/- 109.80
	30	1.38 +/- 0.10	0.43 +/- 0.04	97.48 +/- 17.99	29838.36 +/- 336.28
	40	1.27 +/- 0.08	0.41 +/- 0.03	121.61 +/- 5.47	39658.80 +/- 139.50
	50	1.17 +/- 0.08	0.40 +/- 0.04	155.86 +/- 10.39	49578.65 +/- 131.40
ntEdit k25	15	4.22 +/- 0.04	0.72 +/- 0.05	1.87 +/- 0.15	1563.39 +/- 1.18
	20	4.04 +/- 0.12	0.71 +/- 0.04	2.14 +/- 0.06	1561.03 +/- 0.58
	30	4.17 +/- 0.13	0.70 +/- 0.05	2.92 +/- 0.16	1562.24 +/- 1.11
	40	4.29 +/- 0.17	0.70 +/- 0.04	3.58 +/- 0.44	1562.66 +/- 1.54
	50	4.42 +/- 0.19	0.70 +/- 0.05	4.54 +/- 0.35	1563.29 +/- 1.34
ntEdit k30	15	3.85 +/- 0.15	0.65 +/- 0.07	1.63 +/- 0.09	1567.38 +/- 1.03
	20	3.69 +/- 0.13	0.63 +/- 0.05	2.12 +/- 0.08	1565.45 +/- 0.49
	30	3.73 +/- 0.17	0.62 +/- 0.06	2.71 +/- 0.11	1567.07 +/- 0.78
	40	3.84 +/- 0.19	0.62 +/- 0.05	3.68 +/- 0.07	1566.81 +/- 1.59
	50	3.96 +/- 0.21	0.63 +/- 0.05	4.46 +/- 0.05	1568.74 +/- 0.47
ntEdit k35	15	3.52 +/- 0.28	0.62 +/- 0.07	1.61 +/- 0.02	1570.03 +/- 0.90
	20	3.34 +/- 0.08	0.60 +/- 0.05	1.99 +/- 0.12	1567.67 +/- 0.27
	30	3.27 +/- 0.15	0.58 +/- 0.05	2.67 +/- 0.19	1568.75 +/- 2.27
	40	3.35 +/- 0.15	0.59 +/- 0.05	3.34 +/- 0.14	1569.53 +/- 0.26
	50	3.44 +/- 0.19	0.59 +/- 0.05	4.10 +/- 0.25	1569.12 +/- 1.65
ntEdit k40	15	4.09 +/- 0.48	0.66 +/- 0.08	1.54 +/- 0.04	1571.28 +/- 1.63
	20	3.91 +/- 0.08	0.64 +/- 0.03	1.88 +/- 0.08	1569.42 +/- 1.53
	30	3.62 +/- 0.16	0.60 +/- 0.04	2.50 +/- 0.14	1570.14 +/- 0.44
	40	3.69 +/- 0.15	0.61 +/- 0.05	3.21 +/- 0.16	1570.79 +/- 1.84
	50	3.77 +/- 0.18	0.61 +/- 0.05	3.99 +/- 0.28	1571.92 +/- 0.52
ntEdit k50	15	5.53 +/- 1.31	0.79 +/- 0.15	1.46 +/- 0.08	1570.54 +/- 2.00
	20	5.48 +/- 0.53	0.79 +/- 0.04	1.95 +/- 0.02	1570.67 +/- 1.57
	30	4.00 +/- 0.13	0.65 +/- 0.05	2.43 +/- 0.02	1573.87 +/- 1.25
	40	3.98 +/- 0.17	0.64 +/- 0.05	2.89 +/- 0.13	1574.05 +/- 1.45
	50	4.01 +/- 0.16	0.65 +/- 0.05	3.65 +/- 0.28	1572.71 +/- 0.55
ntEdit k40,k35, k30,k25	15	2.45 +/- 0.17	0.50 +/- 0.05	6.55 +/- 0.26	844.53 +/- 0.12
	20	2.34 +/- 0.15	0.48 +/- 0.04	8.03 +/- 0.16	870.45 +/- 0.09
	30	2.35 +/- 0.18	0.48 +/- 0.04	10.76 +/- 0.19	926.35 +/- 0.18
	40	2.41 +/- 0.19	0.47 +/- 0.04	13.69 +/- 0.42	982.85 +/- 0.07
	50	2.47 +/- 0.22	0.48 +/- 0.04	17.03 +/- 0.76	1039.89 +/- 0.49

Supplemental table 3. QUAST and resource benchmark results for 17-fold coverage controlled *H. sapiens* dataset.

Assembly		Mismatches per 100 kbp	Indels per 100 kbp	Time (min.)	Peak Memory (GB)
Chromosome 1					
Baseline		99.84 +/- 0.02	10.08 +/- 0.00	N/A	N/A
GATK		3.60 +/- 0.04	4.84 +/- 0.05	515.82 +/- 4.66	20.32 +/- 0.27
Pilon		3.81 +/- 0.02	0.48 +/- 0.01	11854.90 +/- 2121.66	170.90 +/- 20.92
Racon		5.12 +/- 0.11	0.66 +/- 0.06	337.32 +/- 39.33	40.06 +/- 0.00
ntEdit	k30	16.26 +/- 0.24	1.64 +/- 0.03	43.07 +/- 3.84	24.02 +/- 0.00
	k40	11.53 +/- 0.08	1.11 +/- 0.02	36.28 +/- 5.77	24.56 +/- 0.00
	k45	9.49 +/- 0.07	0.96 +/- 0.02	37.21 +/- 5.11	24.72 +/- 0.00
	k50	8.62 +/- 0.10	0.91 +/- 0.02	38.39 +/- 8.82	24.67 +/- 0.30
	k55	8.50 +/- 0.09	0.91 +/- 0.02	31.91 +/- 2.40	24.90 +/- 0.00
	k60	8.31 +/- 0.08	0.93 +/- 0.02	29.55 +/- 1.31	24.89 +/- 0.00
	k50,45, 40	8.05 +/- 0.08	0.85 +/- 0.02	102.25 +/- 9.10	23.84 +/- 0.00
Chromosome 21					
Baseline		99.96 +/- 0.30	9.96 +/- 0.06	N/A	N/A
GATK		12.15 +/- 0.17	5.29 +/- 0.12	473.81 +/- 8.76	20.32 +/- 0.27
Pilon		11.95 +/- 0.16	1.38 +/- 0.01	756.28 +/- 72.92	39.77 +/- 0.20
Racon		14.13 +/- 0.09	1.59 +/- 0.04	288.33 +/- 36.80	40.06 +/- 0.00
ntEdit	k30	17.24 +/- 0.05	1.56 +/- 0.03	38.66 +/- 1.59	21.81 +/- 0.00
	k40	12.97 +/- 0.28	1.07 +/- 0.02	34.17 +/- 1.42	23.19 +/- 0.00
	k45	11.18 +/- 0.06	0.97 +/- 0.02	34.48 +/- 0.47	23.57 +/- 0.00
	k50	10.37 +/- 0.19	0.91 +/- 0.01	35.40 +/- 3.43	23.84 +/- 0.00
	k55	10.22 +/- 0.18	0.94 +/- 0.03	32.32 +/- 2.63	23.95 +/- 0.00
	k60	9.78 +/- 0.17	0.94 +/- 0.04	29.81 +/- 1.33	23.86 +/- 0.00
	k70	11.87 +/- 0.12	1.21 +/- 0.05	28.48 +/- 0.03	22.84 +/- 0.00
	k50,45, 40	9.83 +/- 0.17	0.83 +/- 0.01	96.90 +/- 4.67	23.84 +/- 0.00
Genome					
Baseline		100.00 +/- 0.03	10.01 +/- 0.01	N/A	N/A
GATK		3.64 +/- 0.01	4.83 +/- 0.01	1086.93 +/- 10.12	20.32 +/- 0.27
Pilon		N/A	N/A	N/A	N/A
Racon*		5.16 +/- 0.06	0.60 +/- 0.01	1455.60 +/- 131.74	40.06 +/- 0.00
ntEdit	k40	12.07 +/- 0.04	1.08 +/- 0.00	39.99 +/- 9.65	147.10 +/- 24.62
	k45	10.16 +/- 0.02	0.95 +/- 0.00	46.25 +/- 6.11	129.80 +/- 5.26
	k50	9.46 +/- 0.03	0.91 +/- 0.00	38.79 +/- 9.89	149.28 +/- 25.15
	k60	9.47 +/- 0.02	0.96 +/- 0.01	32.06 +/- 2.41	154.79 +/- 18.86

* Racon polishing on the full simulated human genome assembly was performed on each chromosome individually in parallel, and the runtimes were summed.

Supplemental table 4. Performance of polishing utilities on fixing substitutions and indels on simulated *E. coli* genome copies.

Tool	Cov.	k	Sensitivity	Precision	FDR	FPR	Specificity	F1
ntEdit	10	20	0.9711	0.9938	0.0062	0.0011	0.9989	0.9823
		25	0.9691	0.9933	0.0067	0.0011	0.9989	0.9811
		30	0.9572	0.9944	0.0056	0.0010	0.9990	0.9754
		35	0.9497	0.9945	0.0055	0.0010	0.9990	0.9716
		40	0.9317	0.9948	0.0052	0.0010	0.9990	0.9622
		35,30,25	0.9708	0.9940	0.0060	0.0011	0.9989	0.9823
GATK		N/A	0.9785	0.9944	0.0056	0.0011	0.9989	0.9863
Pilon		N/A	0.8780	0.9940	0.0060	0.0010	0.9990	0.9324
ntEdit	15	20	0.9762	0.9938	0.0062	0.0011	0.9989	0.9849
		25	0.9769	0.9934	0.0066	0.0011	0.9989	0.9850
		30	0.9699	0.9943	0.0057	0.0011	0.9989	0.9820
		35	0.9689	0.9942	0.0058	0.0011	0.9989	0.9814
		40	0.9612	0.9945	0.0055	0.0011	0.9989	0.9776
		35,30,25	0.9782	0.9940	0.0060	0.0011	0.9989	0.9860
GATK		N/A	0.9800	0.9944	0.0056	0.0011	0.9989	0.9872
Pilon		N/A	0.9710	0.9940	0.0060	0.0011	0.9989	0.9824
ntEdit	20	20	0.9765	0.9939	0.0061	0.0011	0.9989	0.9851
		25	0.9770	0.9934	0.0066	0.0011	0.9989	0.9851
		30	0.9702	0.9943	0.0057	0.0011	0.9989	0.9821
		35	0.9690	0.9943	0.0057	0.0011	0.9989	0.9815
		40	0.9599	0.9945	0.0055	0.0011	0.9989	0.9769
		35,30,25	0.9785	0.9941	0.0059	0.0011	0.9989	0.9862
GATK		N/A	0.9813	0.9942	0.0058	0.0011	0.9989	0.9877
Pilon		N/A	0.9790	0.9939	0.0061	0.0011	0.9989	0.9863
ntEdit	30	20	0.9765	0.9938	0.0062	0.0011	0.9989	0.9851
		25	0.9769	0.9934	0.0066	0.0011	0.9989	0.9851
		30	0.9706	0.9943	0.0057	0.0011	0.9989	0.9823
		35	0.9703	0.9943	0.0057	0.0011	0.9989	0.9822
		40	0.9643	0.9945	0.0055	0.0011	0.9989	0.9792
		35,30,25	0.9787	0.9941	0.0059	0.0011	0.9989	0.9864
GATK		N/A	0.9820	0.9940	0.0060	0.0011	0.9989	0.9880
Pilon		N/A	0.9811	0.9939	0.0061	0.0011	0.9989	0.9875
ntEdit	40	20	0.9763	0.9938	0.0062	0.0011	0.9989	0.9849
		25	0.9773	0.9934	0.0066	0.0011	0.9989	0.9853
		30	0.9705	0.9943	0.0057	0.0011	0.9989	0.9823
		35	0.9705	0.9943	0.0057	0.0011	0.9989	0.9823
		40	0.9640	0.9945	0.0055	0.0011	0.9989	0.9790
		35,30,25	0.9787	0.9941	0.0059	0.0011	0.9989	0.9864
GATK		N/A	0.9821	0.9942	0.0058	0.0011	0.9989	0.9881
Pilon		N/A	0.9817	0.9938	0.0062	0.0011	0.9989	0.9877
ntEdit	50	20	0.9765	0.9939	0.0061	0.0011	0.9989	0.9851
		25	0.9766	0.9933	0.0067	0.0011	0.9989	0.9849
		30	0.9700	0.9943	0.0057	0.0011	0.9989	0.9821
		35	0.9704	0.9943	0.0057	0.0011	0.9989	0.9822
		40	0.9640	0.9945	0.0055	0.0011	0.9989	0.9790
		35,30,25	0.9787	0.9940	0.0060	0.0011	0.9989	0.9863
GATK		N/A	0.9828	0.9942	0.0058	0.0011	0.9989	0.9885
Pilon		N/A	0.9827	0.9939	0.0061	0.0011	0.9989	0.9883

*NOTE: Mean over 3 runs is shown for all statistics. Racon stats are unavailable as it doesn't output base edit coordinates

Supplemental table 5. Performance of polishing utilities on fixing substitutions and indels on simulated *C. elegans* genome copies.

Tool	Coverage	k	Sensitivity	Precision	FDR	FPR	Specificity	F1
ntEdit	15	25	0.9543	0.9866	0.0134	0.0010	0.9990	0.9702
		30	0.9569	0.9917	0.0083	0.0011	0.9989	0.9740
		35	0.9603	0.9922	0.0078	0.0011	0.9989	0.9760
		40	0.9552	0.9929	0.0071	0.0010	0.9990	0.9737
		50	0.9447	0.9935	0.0065	0.0010	0.9990	0.9685
		40,35,<br>30,25	0.9706	0.9921	0.0079	0.0011	0.9989	0.9812
GATK		N/A	0.9746	0.9938	0.0062	0.0011	0.9989	0.9841
Pilon		N/A	0.9648	0.9937	0.0063	0.0011	0.9989	0.9791
ntEdit	20	25	0.9561	0.9869	0.0131	0.0010	0.9990	0.9713
		30	0.9580	0.9919	0.0081	0.0011	0.9989	0.9747
		35	0.9608	0.9923	0.0077	0.0011	0.9989	0.9762
		40	0.9545	0.9930	0.0070	0.0010	0.9990	0.9734
		50	0.9361	0.9935	0.0065	0.0010	0.9990	0.9640
		40,35,<br>30,25	0.9715	0.9922	0.0078	0.0011	0.9989	0.9817
GATK		N/A	0.9756	0.9937	0.0063	0.0011	0.9989	0.9846
Pilon		N/A	0.9746	0.9937	0.0063	0.0011	0.9989	0.9840
ntEdit	30	25	0.9552	0.9869	0.0131	0.0010	0.9990	0.9708
		30	0.9580	0.9919	0.0081	0.0011	0.9989	0.9747
		35	0.9619	0.9923	0.0077	0.0011	0.9989	0.9769
		40	0.9581	0.9930	0.0070	0.0011	0.9989	0.9752
		50	0.9536	0.9936	0.0064	0.0010	0.9990	0.9732
		40,35,<br>30,25	0.9715	0.9922	0.0078	0.0011	0.9989	0.9817
GATK		N/A	0.9767	0.9936	0.0064	0.0011	0.9989	0.9850
Pilon		N/A	0.9782	0.9937	0.0063	0.0011	0.9989	0.9859
ntEdit	40	25	0.9542	0.9869	0.0131	0.0010	0.9990	0.9702
		30	0.9571	0.9918	0.0082	0.0011	0.9989	0.9742
		35	0.9612	0.9922	0.0078	0.0011	0.9989	0.9764
		40	0.9574	0.9930	0.0070	0.0011	0.9989	0.9749
		50	0.9541	0.9936	0.0064	0.0010	0.9990	0.9735
 ntEdit		40,35,<br>30,25	0.9711	0.9921	0.0079	0.0011	0.9989	0.9815
GATK		N/A	0.9774	0.9936	0.0064	0.0011	0.9989	0.9854
Pilon		N/A	0.9796	0.9937	0.0063	0.0011	0.9989	0.9866
ntEdit	50	25	0.9531	0.9867	0.0133	0.0010	0.9990	0.9696
		30	0.9563	0.9918	0.0082	0.0010	0.9990	0.9737
		35	0.9606	0.9923	0.0077	0.0011	0.9989	0.9761
		40	0.9569	0.9930	0.0070	0.0011	0.9989	0.9746
		50	0.9538	0.9936	0.0064	0.0010	0.9990	0.9733
		40,35,<br>30,25	0.9708	0.9921	0.0079	0.0011	0.9989	0.9813
GATK		N/A	0.9608	0.9926	0.0074	0.0011	0.9989	0.9763
Pilon		N/A	0.9803	0.9938	0.0062	0.0011	0.9989	0.9870

*NOTE: Mean over 3 runs is shown for all statistics. Racon stats are unavailable, as it does not output base edit coordinates

Supplemental table 6. Performance of polishing utilities on fixing substitution and indel on simulated *H. sapiens* chromosomes 1 and 21 and genome copies using 17-fold coverage sequence reads.

Tool	k	Sensitivity	Precision	FDR	FPR	Specificity	F1
Chromosome 1							
ntEdit	30	0.8486	0.9770	0.0230	0.0009	0.9991	0.9083
	35	0.8763	0.9780	0.0220	0.0009	0.9991	0.9244
	40	0.8899	0.9817	0.0183	0.0009	0.9991	0.9335
	45	0.9055	0.9836	0.0164	0.0009	0.9991	0.9429
	50	0.9106	0.9857	0.0143	0.0009	0.9991	0.9466
	55	0.9099	0.9869	0.0131	0.0009	0.9991	0.9468
	60	0.9100	0.9877	0.0123	0.0009	0.9991	0.9473
	70	0.8737	0.9885	0.0115	0.0009	0.9991	0.9276
	50,45, 40	0.9184	0.9841	0.0159	0.0009	0.9991	0.9501
GATK	N/A	0.9349	0.9790	0.0210	0.0009	0.9991	0.9564
Pilon	N/A	0.9491	0.9907	0.0093	0.0010	0.9990	0.9695
Racon	N/A	N/A	N/A	N/A	N/A	N/A	N/A
Chromosome 21							
ntEdit	30	0.8381	0.9770	0.0230	0.0008	0.9992	0.9023
	35	0.8643	0.9775	0.0225	0.0008	0.9992	0.9174
	40	0.8760	0.9813	0.0187	0.0008	0.9992	0.9257
	45	0.8900	0.9829	0.0171	0.0008	0.9992	0.9341
	50	0.8944	0.9850	0.0150	0.0009	0.9991	0.9375
	55	0.8939	0.9861	0.0139	0.0009	0.9991	0.9377
	60	0.8966	0.9869	0.0131	0.0009	0.9991	0.9396
	70	0.8737	0.9874	0.0126	0.0008	0.9992	0.9271
	50,45, 40	0.9042	0.9834	0.0166	0.0009	0.9991	0.9422
GATK	N/A	0.8543	0.9809	0.0191	0.0008	0.9992	0.9132
Pilon	N/A	0.8649	0.9910	0.0090	0.0008	0.9992	0.9237
Racon	N/A	N/A	N/A	N/A	N/A	N/A	N/A
Genome							
ntEdit	40	0.8352	0.9697	0.0303	0.0009	0.9991	0.8974
	45	0.8992	0.9836	0.0164	0.0009	0.9991	0.9396
	50	0.8904	0.9824	0.0176	0.0009	0.9991	0.9341
	60	0.8998	0.9875	0.0125	0.0009	0.9991	0.9416
GATK^	N/A	0.9207	0.9801	0.0199	0.0010	0.9990	0.9478
Pilon	N/A	N/A	N/A	N/A	N/A	N/A	N/A
Racon	N/A	N/A	N/A	N/A	N/A	N/A	N/A

*NOTE: Mean over 3 runs is shown for all statistics. Racon stats are unavailable, as it does not output base edit coordinates

^Stats averaged across all chromosomes

Supplemental table 7. Suggested ntEdit options for polishing Illumina and SMS* assemblies with Illumina reads

	ntEdit parameters				
Genome size	k	i	d	x	y
<10 Mbp	25	≤4	≤5	5	9
10-500 Mbp	35	≤4	≤5	5	9
100-3000 Mbp	40-50[1]	≤3	≤3	5	9
>3 Gbp	50	≤3	≤3	5	9

***SMS: Single-molecule sequencing**

[1]A lower k (ie. k40) may work best with a more erroneous assembly (see Table S8), and a larger k (ie. k50) with a more accurate one. We recommend running ntEdit iteratively with different k values for best results. We do not recommend setting the indel options (–i and –d) too high on large genomes, especially if they are assembled from low indel Illumina reads

Note: These are suggestions based on tests reported herein. We encourage users the explore a wide range of options, which may work best with their data

Supplemental table 8. BUSCO* analysis on NA12878 human assemblies, pre and post polishing with 54X Illumina reads.

Assembly[1]	Comparator	#Edits (M)	Run time hh:mm[2]	Peak RAM (GB)	Complete BUSCO[3] (%)	Frag-mented BUSCO	Missing BUSCO
**PacBio**	Baseline	N/A	N/A	N/A	5285 (85.4)	458	449
(Pendleton et al., 2015)	ntEdit						
	k40 i1 d1	3.52	2:10	99.6	5650 (91.2)	261	281
N50 = 25.41 Mbp	k40 i2 d2	3.61	2:10	96.8	5650 (91.2)	262	280
	k40 i3 d3	3.63	2:10	97.9	5651 (91.3)	263	278
	k50 i1 d1	3.44	2:10	87.4	5643 (91.1)	258	291
	k50 i2 d2	3.53	2:10	98.2	5647 (91.2)	259	286
	k50 i3 d3	3.54	2:10	97.6	5647 (91.2)	258	287
	GATK	2.66	42:21	38.0	5285 (85.4)	460	447
	Racon[4]	N/A	40:55	21.0	5670 (91.6)	243	279
**Nanopore**	Baseline	N/A	N/A	N/A	5647 (91.2)	274	271
(Jain et al., 2018)	ntEdit						
	k40 i1 d1	0.79	2:10	41.3	5662 (91.4)	262	268
N50 = 7.67 Mbp	k40 i2 d2	1.13	2:10	42.5	5663 (91.5)	258	271
	k40 i3 d3	1.29	2:10	46.8	5669 (91.6)	256	267
	k50 i1 d1	0.59	2:10	40.8	5663 (91.5)	262	267
	k50 i2 d2	0.84	2:10	41.9	5673 (91.6)	250	269
	k50 i3 d3	0.95	2:10	44.4	5670 (91.6)	250	272
	GATK	0.96	41:45	38.0	5654 (91.3)	270	268
	Racon[4]	N/A	45:54	18.0	5681 (91.7)	234	277
**Supernova**[5]	Baseline	N/A	N/A	N/A	5722 (92.4)	228	242
	ntEdit						
N50 = 44.06 Mbp	k40 i1 d1	0.08	2:20	133.8	5725 (92.5)	225	242
	k40 i2 d2	0.08	2:18	133.9	5725 (92.5)	225	242
	k40 i3 d3	0.09	2:18	133.9	5725 (92.5)	225	242
	k50 i1 d1	0.09	2:19	134.4	5723 (92.4)	228	241
	k50 i2 d2	0.09	2:18	134.4	5724 (92.4)	226	242
	k50 i3 d3	0.10	2:18	134.0	5724 (92.4)	226	242
	GATK	0.19	45:05	39.0	5724 (92.4)	226	242
	Racon[4]	N/A	44:31	11.0	5722 (92.4)	224	246

[1]The NA12878 assemblies are discussed in detail in Watson and Warr (2019). N/A: Not Available.

[2] Run times are indicated for the full tool pipeline (includes read indexing, alignments, Bloom filter creation, where applicable). ntHits ran in 2h08m and 2h15m and required 36.9 and 38.5 GB RAM to process 322.4M Illumina read pairs (2x251bp, ~54X coverage of human genome) at k40 and k50, respectively. The ntEdit runs completed within 2-4 minutes

[3]BUSCO resources: Lineage, euarchontoglires_odb9 (eukaryote), Number of BUSCO searched: 6192

[4]Racon polishing was run in parallel by splitting each assembly into 20 partitions, and polishing each partition separately (see supplemental methods text). Runtimes are summed over all partitions

[5]Assembly from: https://support.10xgenomics.com/de-novo-assembly/datasets/2.1.0/wfu
